# Supplementary material for: Seminoma and Embryonal Carcinoma Footprints Identified by Analysis of Integrated Genome-Wide Epigenetic and Expression Profiles of Germ Cell Cancer Cell Lines
Source: PLoS One. 2014 Jun 2;9(6):e98330. doi: 10.1371/journal.pone.0098330 (PMC4041891; doi:10.1371/journal.pone.0098330)
Supplement: File S1 — ZIP file containing DMRforPairs output for significant regions. Please start from the html files. (ZIP) [file pone.0098330.s008.zip › figures/3362.pdf]

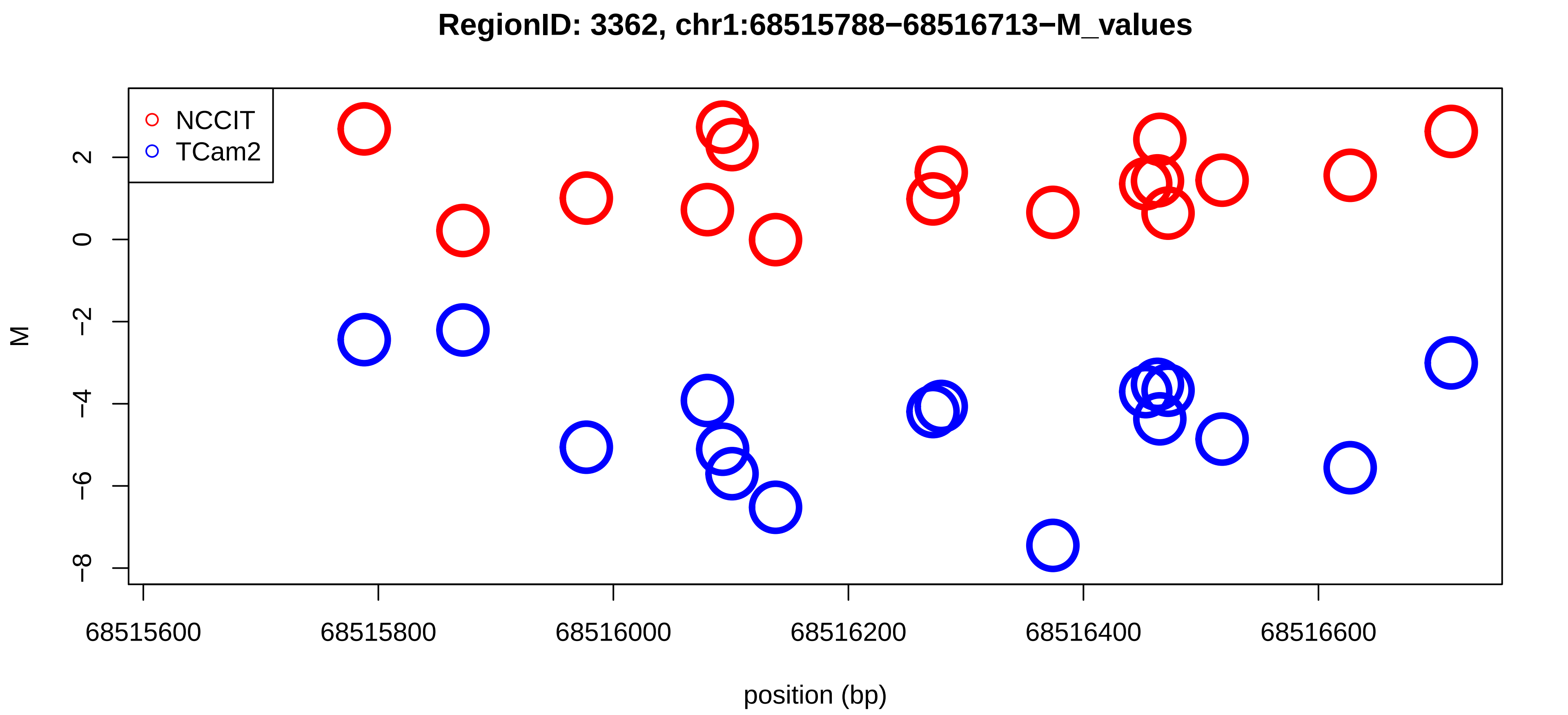

RegionID: 3362, chr1:68515788−68516713−M_values
NCCIT
TCam2
M
2
0
−2
−4
−6
−8
68515600
68515800
68516000
68516200
68516400
68516600
position (bp)

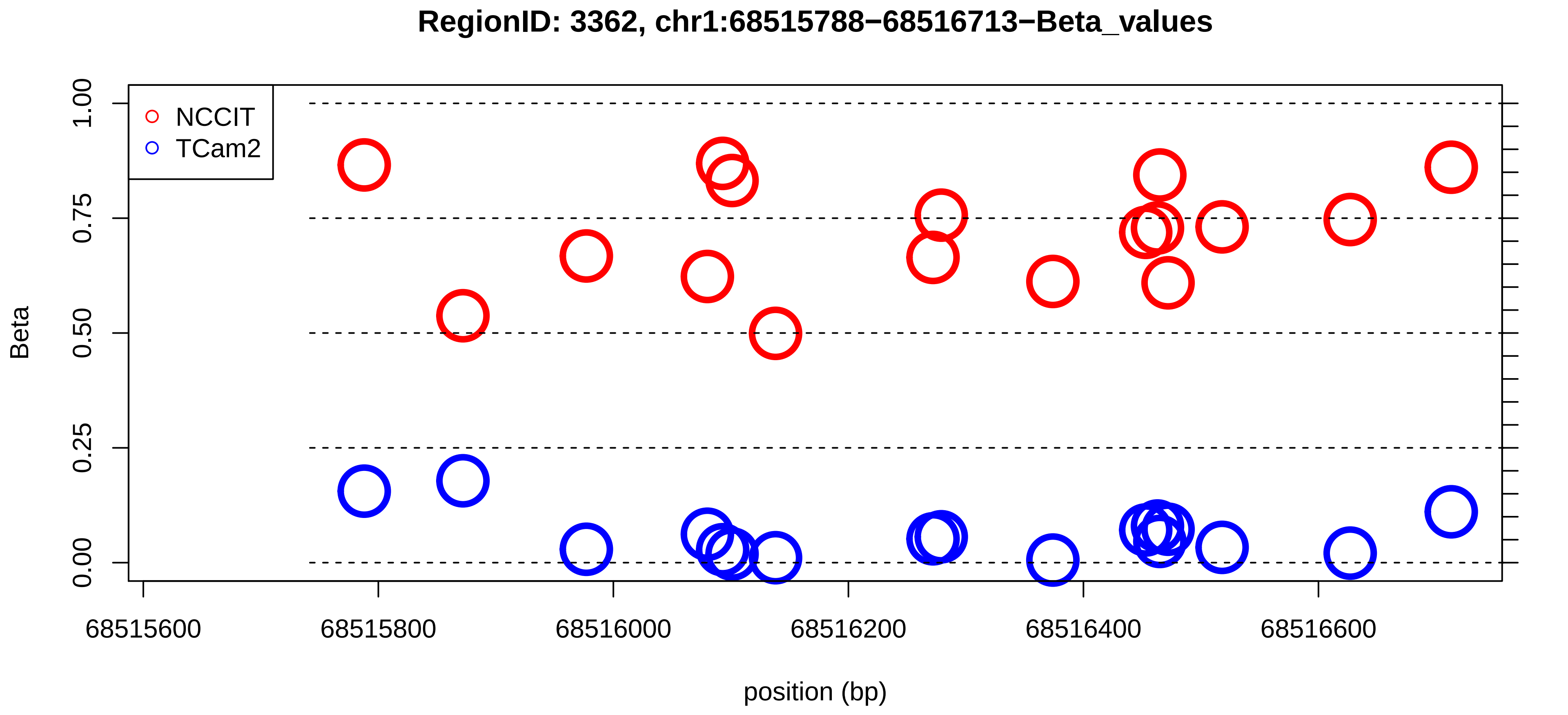

RegionID: 3362, chr1:68515788−68516713−Beta_values
NCCIT
TCam2
Beta
1.00
0.75
0.50
0.25
0.00
68515600
68515800
68516000
68516200
68516400
68516600
position (bp)

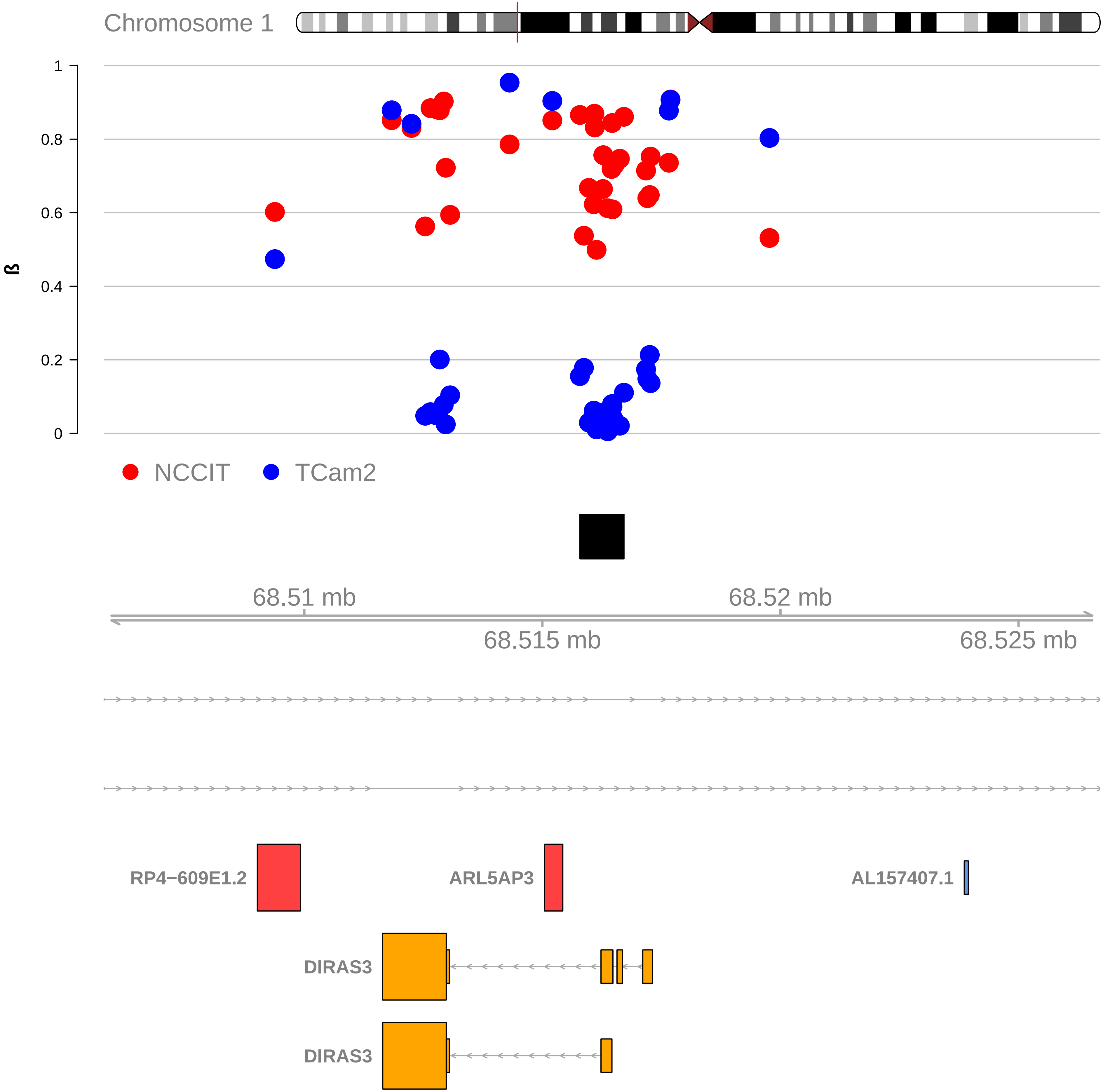

Chromosome 1
1
0.8
0.6
0.4
0.2
0
ß
NCCIT
TCam2
68.51 mb
68.52 mb
68.515 mb
68.525 mb
RP4−609E1.2
ARL5AP3
AL157407.1
DIRAS3
DIRAS3
